# Research focus and emerging trends of cancer-related fatigue in nursing arena: A bibliometric analysis from 2012 to 2021

**DOI:** 10.1097/MD.0000000000040405

**Published:** 2024-11-15

**Authors:** Rong Zheng, Xi Chen, Xiuzhi Xu, Yongxia Song, Xiaodi Ju, Wenru Wang, Jingfang Hong

**Affiliations:** a School of Nursing, Anhui Medical University, Hefei, China; b Cangzhou Central Hospital, Cangzhou, China; c Nursing Department, No.2 People’s Hospital of Fuyang City, Anhui Province, China; d Alice Lee Centre for Nursing Studies, Yong Loo Lin School of Medicine, National University of Singapore, Singapore.

**Keywords:** bibliometric analysis, cancer-related fatigue, CiteSpace, nursing

## Abstract

**Background:**

To explore distributed characteristics and identify research focus and emerging trends related to cancer-related fatigue (CRF) in the nursing field.

**Methods:**

Data were collected from the Web of Science Core Collection database between January 2012 and December 2021 using an advanced search strategy. Data were extracted and analyzed using CiteSpace and Microsoft Excel.

**Results:**

A total of 967 articles were included in this study. The number of published nursing studies on CRF has increased with slight fluctuations. Keyword co-occurrence analysis and timeline view indicated that CRF is closely related to quality of life, and there is a correlation between CRF and other symptoms. Moreover, increasing attention has been paid to CRF nursing interventions. The assessment tools and their different language versions, risk factors and reviews of CRF were the research frontiers in recent years with citation bursts.

**Conclusions:**

In the field of nursing, the focus of CRF research is still on risk factors, adverse outcomes and nursing management. Assessment tools will continue to be developed and additional risk factors will be studied in the future.

## 1. Introduction

Cancer, a significant public health issue, has emerged as one of the leading causes of disability and mortality globally.^[1]^ According to the 2020 Global Cancer Statistics report, it is estimated that there were approximately 19.3 million new cancer cases and nearly 9.96 million deaths globally.^[2]^ A majority of these patients, who underwent conventional treatments such as surgery, radiation, or systemic therapy, experienced a range of cancer-related symptoms and treatment side effects.^[3]^ Cancer-related fatigue (CRF) is recognized as one of the most prevalent symptoms throughout the cancer journey, accounting for 62% of the reported symptoms in cancer patients.^[4]^ As defined by the National Cancer Comprehensive Network, CRF is “a distressing, persistent, subjective sense of physical, emotional and/or cognitive tiredness or exhaustion related to cancer or cancer treatment, which is not proportional to recent activity and interferes with usual functioning.”^[5]^ Unlike fatigue resulting from excessive exercise or lack of sleep, CRF is more severe and persistent, and is less likely to be alleviated by rest.^[6]^ Given the high prevalence of these symptoms, it is imperative for nurses, who have frequent contact with patients, to recognize and address CRF in their care.

The term ‘CRF’ was initially introduced in 1979 by Haylock and Hart.^[7]^ Subsequently, in 1986, Piper defined CRF from a nursing standpoint as “subjective fatigue associated with cancer, characterized by variable duration and intensity that is not proportional to or not alleviated by individual action.”^[8]^ Due to its high prevalence and prolonged duration, CRF was incorporated into the guidelines issued by National Cancer Comprehensive Network.^[5]^ As one of the most common symptoms of cancer, CRF arises from the interplay of multiple factors, including disease-related elements, treatment-related side effects and psychosocial influences, throughout the whole process of tumor occurrence, progression, treatment and prognosis.^[9,10]^ CRF imposes substantial physical and psychological strain on patients, disrupting their physiological functions and overall quality of life.^[11]^ Given the absence of a specific drug for CRF treatment, researchers have begun to work on its mechanisms to devise interventions that could mitigate symptoms and augment quality of life. The mechanisms of CRF remain under investigation, with the pathogenetic mechanism being the most widely recognized.^[12]^ Research has demonstrated that interventions such as massage therapy,^[13]^ psychosocial support,^[14]^ nutritional counseling^[15]^ and cognitive behavioral therapy for sleep^[16]^ may effectively mitigate cancer-related fatigue (CRF).

Bibliometric is a visualized atlas that quantitatively analyzes the knowledge of a discipline by combining mathematical and statistical methods. This tool facilitates the identification of popular topics and emerging trends across various disciplines through keyword and co-citation analyses of existing studies.^[17]^ Several software applications have been developed for this purpose, including CiteSpace, VOSviewer, and Gephi and Pajek.^[18]^ CiteSpace, a Java-based visualization software developed by Professor Chen Chaomei,^[19]^ is particularly useful in analyzing and visualizing the trends and patterns of scientific literature. It presents the structure and distribution of scientific knowledge, with a particular focus on identifying key points in the development of a field, such as turning points and key knowledge acquisition points.^[20]^ The number of literature using traditional methods such as SPSS and Pajek is limited, thereby posing challenges for researchers attempting to analyze large volumes of studies. In contrast, CiteSpace offers the advantage of unlimited article inclusion. This software can yield precise and lucid results within a remarkably short timeframe when applied to hundreds to thousands of articles. Within the field of nursing, CiteSpace has been widely adapted to analyze citation counts and collaborations across countries, institutions, journals, and authors. It also aids in identifying keyword trends related to specific topics.^[21]^ The application of CiteSpace in analyzing existing studies holds significant implications for nurses involved in cancer management and symptom nursing.

The volume of published studies on CRF has seen a significant surge over the years. However, to the best of our knowledge, no bibliometric studies of published articles on CRF in the field of nursing have been reported. This study aimed to visually present the research focus and emerging trends of CRF through visualizations analyzed using CiteSpace. The insights garnered from this study will help researchers to gain a comprehensive understanding of the current research hotspots, trends and frontiers in CRF.

## 2. Methods

### 2.1. Design

The publications on CRF studies in nursing journals, from 2012 to 2021 were identified and analyzed using descriptive bibliometric analysis.

### 2.2. Data collection and search strategy

Literature retrieval was performed using the Web of Science Core Collection database on March 7, 2022. The research strategy was set to “((TS = cancer* OR TS = neoplasm* OR TS = tumor* OR TS = malignanc*) AND (TS = fatigue OR TS = lassitude OR TS = tired OR TS = exhaust*) AND (SU = Nursing)) NOT (TS = burnout).” Language was limited to “English.” The publication type was set to “article OR review.” The publication time was limited from January 1, 2012 to December 31, 2021. The documents with incomplete keywords or abstracts were excluded. A total of 967 articles were included in the study.

### 2.3. Data analysis

CiteSpace 5.8.R3 was used to perform statistical analysis, visualize the structure and distribution of research fields to calculate annual published articles, identify collaborative networks among countries, institutions, journals and authors, analyze co-occurring keywords and burst terms and perform co-citation analysis. Microsoft Excel was used to record the data output from CiteSpace.

The parameters for CiteSpace were set as follows: “Year per slice” for 1 year; “Term source” including title, abstract, author keywords and keywords plus; “Top 50 levels” was selected as the most cited or occurred items from each slice; and “k = 25” was selected to use a modified g-index in each slice; “Node types” and “Pruning” were set based on specific conditions. For instance, during a keyword co-occurrence analysis, “Node types” was set to “Keyword” and “Pruning” was set to “Pruning sliced networks” and “Pathfinder” to highlight the core structure.

### 2.4. Ethical considerations

Ethical approval was not required as this study was conducted without the direct involvement of human or animal subjects.

### 2.5. Validity and reliability

All citation data were exported in plain text format from the Web of Science core database, encompassing complete records and cited references. The plain text file was named “Download.” The data were independently analyzed by 2 researchers. In instances of discrepancy, a discussion between the researchers took place, with the final decision being made by the corresponding author. All analyses were based on quantitative data, ensuring the reliability of the results. Two values are generated from the clustering result of a timeline view. A modularity Q value exceeding 0.3 indicates the presence of a significant clustering structure, while a silhouette value surpassing 0.5 signifies a reasonable clustering outcome.

## 3. Results

### 3.1. Analysis of annual publications

**Figure 1.**
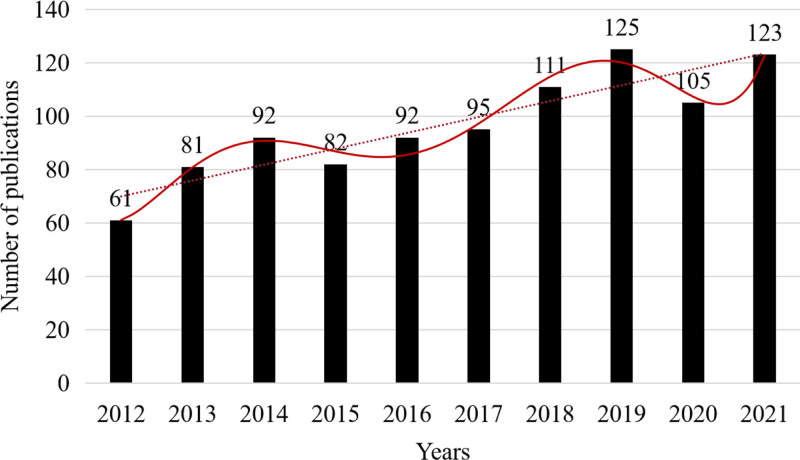
Number of cancer-related fatigue nursing research in Web of Science from 2012 and 2021.

Figure 1 shows the annual trends of CRF-related publications in nursing journals from 2012 to 2021. After excluding documents with incomplete keywords and abstracts, a total of 967 articles were published and indexed on the Web of Science, including 855 articles (88.4%) and 112 reviews (11.6%). With the exception of 2 downward trends in 2015 and 2020, there was a consistent increase in the number of publications steadily increased over time. This suggests that the topic of CRF has progressively garnered the interest of nursing scholars.

### 3.2. Analysis of countries/regions and institutions

**Figure 2.**
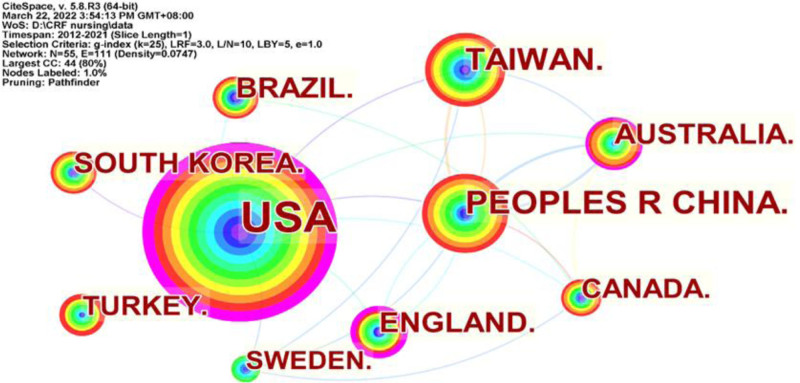
Research countries/regions in Web of Science from 2012 to 2021. *Note*: The size of a circle is proportional to the number of articles published in a country/region. The color of rings of a circle is corresponding to the year. The purple rings of circle indicate high betweenness centralities. The links between nodes represent cooperation between countries/regions.

Figure 2 illustrates the existing international collaboration networks among countries/regions in CRF-related studies in nursing journals. This analysis yielded a network comprising 55 nodes and 111 links, where the nodes symbolize the number of countries/regions and the links denote cooperative associations between them. The top 5 countries/regions were the USA (N = 427), the People’s Republic of China (N = 85), Taiwan China (N = 70), South Korea (N = 47) and Australia (N = 46), which suggests that the USA and China were the leading force in nursing research on CRF. The top 5 countries/regions in terms of centrality were the USA (Centrality = 0.60), England (Centrality = 0.25), Australia (Centrality = 0.12), Netherlands (Centrality = 0.10) and Sweden (Centrality = 0.09), which signifies they had close collaboration with other countries.

**Figure 3.**
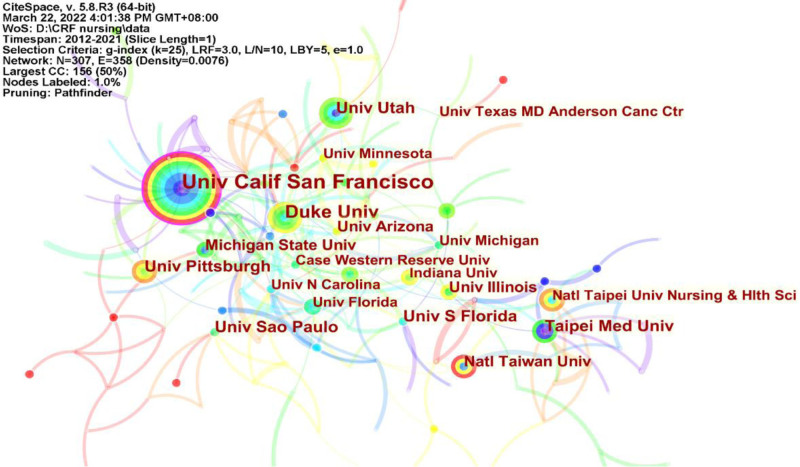
Research institutions in Web of Science from 2012 to 2021. *Note*: The size of a circle is proportional to the number of articles published in an institution. The color of rings of a circle is corresponding to the year. The purple rings of circle indicate high betweenness centralities. The links between nodes represent cooperation between institutions.

Figure 3 illustrates the collaborative visualization networks among participating institutions in the CRF nursing research. A total of 307 institutions contributed to this study. The 5 most productive universities were the University of California, San Francisco (N = 38), Duke University (N = 25), the University of Utah (N = 18), Taipei Medical University (N = 17), and the University of Pittsburgh (N = 17). Notably, 4 of these top 5 institutions are based in the USA, indicating a leading role for the USA in CRF nursing research. The University of California, San Francisco exhibited the highest centrality (Centrality = 0.14), suggesting it had the most extensive collaboration with other institutions.

### 3.3. Analysis of journals and co-cited journals

Table 1 presents a comprehensive list of the top 10 most popular journals and co-cited journals for CRF study publications, along with their total respective publication counts and journal impact factors. The Cancer Nursing journal emerged as the most prolific in terms of published articles, succeeded by the European Journal of Cancer Care and the European Journal of Oncology Nursing. Notably, 7 of these highly productive journals achieved an official impact factor exceeding 2.0 in 2021. In terms of publisher representation, 6 were based in the USA, 3 in England and 1 in India.

**Table 1:** Research journals and co-cited journals in Web of Science from 2012 to 2021.

Journal	Count	IF2021	Co-cited journal	Count	IF2021
Cancer Nursing	148	2.760	Supportive Care in Cancer	596	3.359
European Journal of Cancer Care	136	2.328	Journal of Pain and Symptom Management	554	5.576
European Journal of Oncology Nursing	112	2.588	Oncology Nursing Forum	553	1.803
Oncology Nursing Forum	79	1.803	Journal of Clinical Oncology	546	50.717
Clinical Journal of Oncology Nursing	64	1.283	Cancer Nursing	506	2.760
Journal of Clinical Nursing	47	4.423	Cancer	506	6.921
Biological Research for Nursing	38	2.318	Psycho-Oncology	446	3.955
Journal of Pediatric Oncology Nursing	28	1.966	European Journal of Oncology Nursing	330	2.588
Seminars in Oncology Nursing	22	3.527	European Journal of Cancer Care	262	2.328
Asia Pacific Journal of Oncology Nursing	20	2.22	Quality of life research	234	3.44

IF2021, 2021 journal impact factor.

When ranked according to the total citation frequency, Supportive Care in Cancer was first, and Journal of Pain and Symptom Management and Oncology Nursing Forum were second and third, respectively. Amongst the top 10 highly cited journals, 5 were from the USA, 2 were from England, 2 were from the Netherlands and 1 was from Germany.

### 3.4. Analysis of authors and co-cited authors

The top 10 authors and co-cited authors are listed in Table 2. Professor M Christine, from the University of California, San Francisco, is devoted to this research area and has published 26 articles, which is the most common among the authors. SM Paul and A Dhruva are second and third, respectively. The most co-cited author was JE Bower with 135 citations. Interestingly, more than half of the top ten authors and co-cited authors were from the USA.

**Table 2:** Research authors and co-cited authors in Web of Science from 2012 to 2021.

Authors	Count	Nationality	Co-cited authors	Count	Nationality
Christine M	26	USA	Bower JE	135	USA
Paul SM	15	USA	Berger AM	109	USA
Dhruva A	10	USA	Christine M	90	USA
Shun S	9	China	Cella D	82	USA
Conley YP	8	USA	Cleeland CS	79	USA
Hooke MC	8	USA	Kim HJ	74	Korea
Aouizerat BE	8	USA	Aaronson NK	70	Netherlands
Dunn LB	6	USA	Wang XS	67	USA
West C	6	USA	Barsevick AM	62	England
Baggott C	5	USA	Zigmond AS	61	England

### 3.5. Analysis of research hotspots, trends and frontiers with keywords

**Figure 4.**
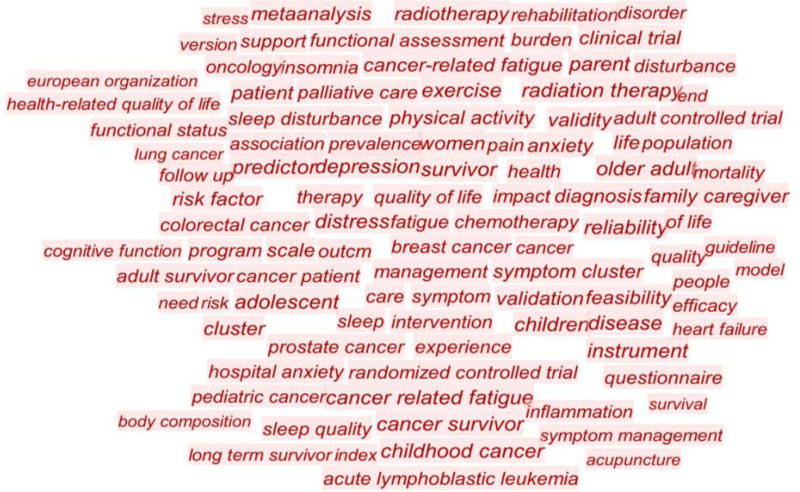
Keywords of cancer-related fatigue nursing research from 2012 to 2021. *Note*: The larger the font size of keywords, the more frequently they appear in the literature.

Hotspots in the field of CRF research and their evolution were extracted using CiteSpace. A total of 394 keywords were identified and are shown in Figure 4. The 3 keywords with the highest frequency were quality of life (495 times), fatigue (257 times), and breast cancer (257 times). The keywords “survivor” and “women” were also used frequently. These keywords collectively signify significant focal points for CRF in the field of nursing.

**Figure 5.**
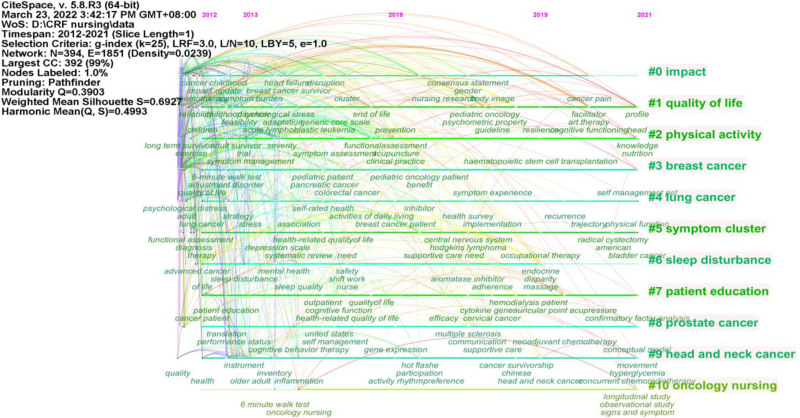
Keywords timeline view of cancer-related fatigue nursing research from 2012 to 2021. *Note*: Q, Modularity. When Q is >0.3, it indicates that the cluster structure is stable. S, Silhouette. When S is greater than 0.5, it indicates that the cluster is reasonable.

Figure 5 presents a timeline view of the top 11 largest clusters of citing articles on CRF in the field of nursing. These include impact, quality of life, physical activity, breast cancer, lung cancer, symptom cluster, sleep disturbance, patient education, prostate cancer, head and neck cancer, and oncology nursing. In the earlier studies on CRF in the field of nursing, factors influencing CRF have received widespread attention. These studies focused on patients with breast cancer, lung cancer, prostate cancer and head and neck cancer. Previous studies have found a correlation between CRF and sleep disturbances. Building upon this foundation, subsequent research has increasingly emphasized fatigue-related symptom clusters. In recent years, there has been a shift towards exploring nursing interventions for patients with CRF, the relationship between CRF and quality of life, and physical activities-based interventions for CRF. This progression provides a clearer understanding of the evolution of CRF research more clearly over time.

**Figure 6.**
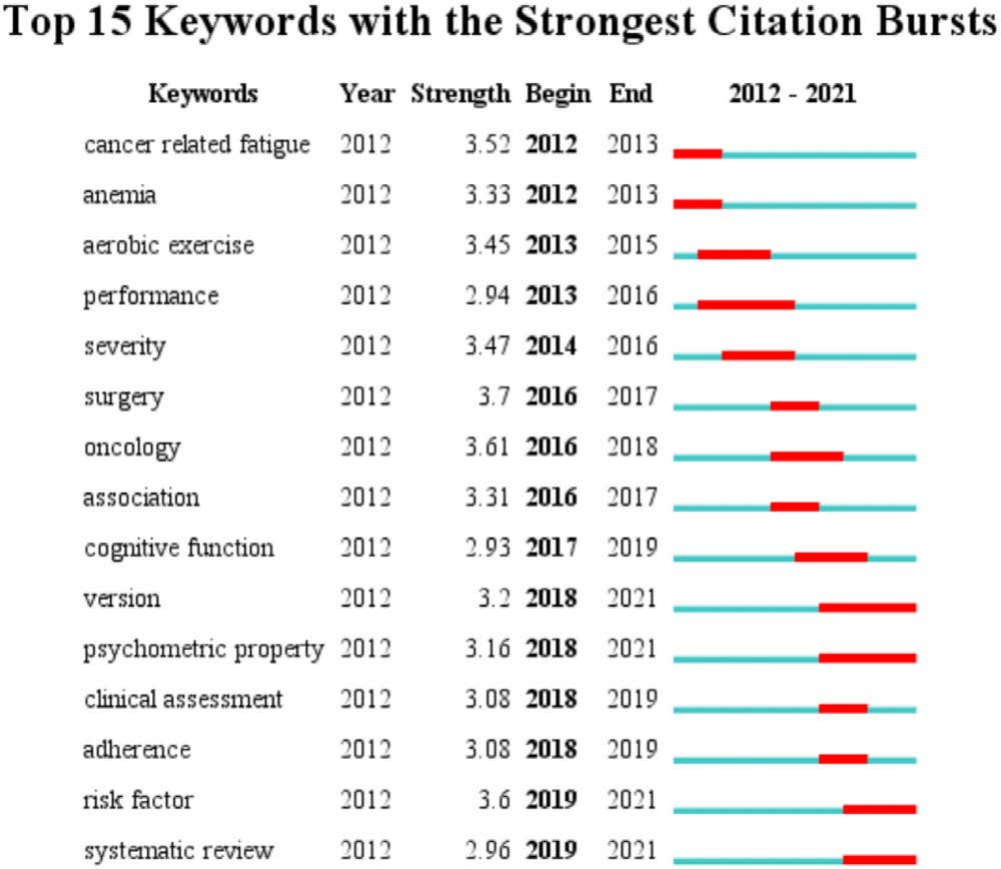
Keywords with strongest citation bursts of cancer-related fatigue nursing research from 2012 to 2021. *Note*: The occurrence periods of burst keywords are shown in red.

Figure 6 shows the top 15 keywords with citation bursts. The top 5 keywords for burst strength were “surgery,” “oncology,” “risk factor,” “cancer-related fatigue” and “severity.” Studies based on these keywords represent research frontiers at a given time. Keywords with citation bursts in recent years are listed as follows: “version,” “psychometric property,” “risk factor” and “systematic review.” This result shows that CRF assessment tools and their different language versions, and risk factors will likely continue to be the research frontiers in the coming years. In addition, reviews of CRF will also be the emerging trend of research in the nursing field over the next few years.

### 3.6. Analysis of articles co-citation

Table 3 presents the top ten cited articles on CRF in the nursing field. Among the top 10 publications, there were 4 reviews and 6 clinical studies. These articles mainly focused on symptom clusters associated with CRF, its causes and its impact on quality of life. The article with the highest number of citations, titled “Symptom burden and quality of life in survivorship a review of the literature,” was published in the Cancer Nursing journal in 2015. This study found that the symptom burden of cancer patients negatively affects their quality of life. Of the top 10 highly cited articles, 6 were from the USA, others were from the Netherlands, Italy, and South Korea, and 1 was a collaboration between the USA and South Korea.

**Table 3:** Top 10 cited articles for cancer-related fatigue nursing research.

TC	Author	Year	Title	Journal
143	Wu HS (USA)	2015	Symptom burden and quality of life in survivorship a review of the literature	Cancer Nursing
112	Custers JAE (USA)	2014	The cancer worry scale detecting fear of recurrence in breast cancer survivors	Cancer Nursing
93	Mishra SI (USA)	2014	Are exercise programs effective for improving health-related quality of life among cancer survivors? a systematic review and meta-analysis	Oncology Nursing Forum
86	Doong SH (USA)	2015	Associations between cytokine genes and a symptom cluster of pain, fatigue, sleep disturbance, and depression in patients prior to breast cancer surgery	Biological Research for Nursing
85	Albrecht TA (USA)	2012	Physical activity in patients with advanced-stage cancer: a systematic review of the literature	Clinical Journal of Oncology Nursing
83	Biglia N (Italy)	2012	Objective and self-reported cognitive dysfunction in breast cancer women treated with chemotherapy: a prospective study	European Journal of Cancer Care
80	Moody K (USA)	2013	Helping the helpers: mindfulness training for burnout in pediatric oncology-a pilot program	Journal of Pediatric Oncology Nursing
78	Lee MK (Korea)	2014	A Web-based self-management exercise and diet intervention for breast cancer survivors: pilot randomized controlled trial	International Journal of Nursing Studies
77	Kestler SA (USA)	2012	Review of symptom experiences in children and adolescents with cancer	Cancer Nursing
74	Kim HJ (Korea)	2012	Common biological pathways underlying the psychoneurological symptom cluster in cancer patients	Cancer Nursing

TC, total citation, which can be available directly when using CiteSpace for article analysis.

## 4. Discussion

This research undertook a comprehensive bibliometric analysis of CRF studies in the nursing field, utilizing CiteSpace to identify the most prolific countries, institutions, journals and authors contributing to CRF research in nursing journals. The objective was to investigate the prevailing research hotspots, trends and frontiers of CRF. In this investigation, we employed bibliometric analysis was used to visualize 967 studies on CRF in the nursing field, which were indexed in the Web of Science Core Collection database from 2012 to 2021. The findings offer researchers an exhaustive understanding of the nursing field of CRF, thereby providing a direction for subsequent research.

As our a greater understanding of diseases and living standards has advanced, the symptom burden in cancer patients has become a focal point for nursing research. This study found a fluctuating increase in the number of CRF publications in the field of nursing over the past decade. This pattern may be attributed to the high prevalence and severity of CRF in cancer patients. The manifestation of CRF in cancer patients is influenced by the disease itself, with up to 40% of patients reporting fatigue at the time of cancer diagnosis. Treatment methods is also significantly contribute to CRF, affecting 80%-90% of cancer patients undergoing chemotherapy or radiation therapy.^[6]^ This observation is further corroborated by the fact that the incidence of CRF patients stands at 74.4%, with 36.9% reporting moderate-to-severe fatigue.^[22]^ Developed countries, particularly the USA, have established a leading role in CRF nursing research. This assertion is supported by our findings, as the USA accounted for 38.2% of global articles published on this topic. Four of the top 5 institutions publishing articles on CRF were from the USA, and 9 of the top ten authors were also from the USA, which published the most highly cited articles. The USA has consistently outperformed other countries in CRF research, being the first country to investigate CRF and developing numerous scales to measure symptom burden, thereby setting a precedent for future research.^[23]^ Among developing countries, China has emerged as a significant contributor in recent years. With a large population suffering from cancer, including those experiencing CRF, China’s contribution to CRF research is substantial. However, research on CRF in other developing countries remains limited. In low- and middle-income countries, clinicians often underestimate CRF in cancer patients during treatment.^[24]^ The University of California, San Francisco, exhibits the highest intermediary centrality, indicating its close association with other schools involved in CRF research. The institution primarily concentrates on the etiological mechanisms of CRF and its correlation with other symptoms.

The analysis of subject journals contributes to understanding the direction of a particular research topic.^[25]^ Ten journals accounted for 71.8% of the publications on CRF research, suggesting that these journals were active and dominant in the nursing field of CRF. Thus, these journals serve as primary conduits for information transmission and communication platforms for pivotal research in this field. Notably, over 3-fifths of the top ten publications ranked within the upper 50% of all nursing journals, as per the impact factors updated in 2021. For articles related to CRF, it is advisable to select journals with a higher number of related articles and appropriate influencing factors should be selected for publication, especially within nursing journals.

Document co-citation analysis serves as a tool to bolster interdisciplinary research by assisting scholars in identifying peer-recognized academic documents and augmenting the breadth of pertinent studies.^[26]^ By conducting author and journal co-citation networks, we identified key authors and journals in CRF. The analysis found that JE Bower has emerged as the most frequently cited authoritative expert on CRF in the nursing field. Concurrently, M Christine was not only among the top ten prolific authors but also among the top ten co-cited authors, suggesting her significant contribution to CRF in the nursing field with considerable influence and heightened attention. The majority of her studies she centered on the characteristics and influential factors of morning and evening fatigue in cancer patients. The 2 highest-cited journals, Supportive Care in Cancer and Journal of Pain and Symptom Management, received the largest number of co-cited references. Both journals focused on supportive treatment and symptom care as alternatives or supplements to disease management during the initial stages of cancer treatment, underscoring their pivotal roles in CRF nursing research. Additionally, our study also found that “Symptom burden and quality of life in survivorship a review of the literature” written by Wu and Harden as the most frequently cited article, concluding that the symptom burden of cancer patients is substantial and detrimental to their quality of life. It recommended that longitudinal studies with long-term follow-up should be conducted to understand the negative effects of symptom burden in the long term. A theoretical framework for CRF research is also presented in this study.^[27]^

In order to elucidate the relationship between research topics and hotspots, we undertook a keyword co-occurrence analysis. The 3 most frequently occurring keywords with the highest frequencies were “quality of life,” “fatigue” and “breast cancer,” which represent the research hotspots of CRF in the nursing field. It is noteworthy that these keywords frequently appear in tandem and exhibit significant interrelatedness. As defined, CRF pertains the fatigue experienced by cancer patients. Predominantly, studies have been conducted on patients with breast cancer. Beyond the disease itself, these patients are subjected to various treatments such as surgery, chemotherapy, or radiation therapy.^[28]^ Consequently, they endure a spectrum of cancer-related symptoms, including CRF. Recent research indicates that CRF significantly impacts the quality of life of cancer patients, encompassing not only breast cancer patients but also others.^[29]^ Therefore, future endeavors to enhance the quality of life of cancer patients should prioritize mitigating the degree of CRF.

The temporal analysis of keyword clustering provides insights into the evolution and trends of the research field. Through a comprehensive timeline visualization, our study identified numerous keywords that have gained prominence in recent years, indicative of the swift advancements in CRF in the nursing field. The research hotspots and emerging trends can be divided into 4 categories as follows:

Firstly, patients with different cancer types suffered from CRF. CRF is common and heterogeneous among patients with different types of cancers. According to visualization analysis, CRF has been the subject of extensive research in patients with lung, prostate, and head and neck cancers, especially breast cancer. This finding aligns with a meta-analysis conducted by Al Maqbali et al (2021), which found that the majority of studies included patients with breast cancer, lung cancer, and prostate cancer. Notably, 49.7%, 48.8%, and 26.3% of these studies reported CRF respectively.^[30]^ This may explain the significance of high prevalence of CRF, underscoring the need for close monitoring by nurses.

Secondly, adverse patient outcomes were influenced by CRF. The majority of research has focused on the relationship between CRF and quality of life. Patients often encounter CRF during both the treatment and rehabilitation phases, which can adversely affect their social, psychological and physiological functions, thereby diminishing their overall quality of life.^[31]^ It is observed that an increase in CRF levels corresponds to a decline in quality of life, and fluctuations in CRF over time can serve as reliable predictors of quality of life.^[32]^ Furthermore, it has been established that cancer patients do not solely experience the symptom of CRF. Instead, they typically encounter symptom clusters that include pain, sleep disturbances, depression and so on, which collectively impact their the quality of life.^[33,34]^

Thirdly, many risk factors affect CRF. Although the mechanism of CRF remain elusive, research has indicated that CRF may be influenced by demographic, clinical, physiological and psychological factors. Chemotherapy can induce gastrointestinal, hematological, and neurotoxicity, potentially contributing to CRF as a common symptom.^[35]^ This notion is corroborated supported by a study of exploring the symptom trajectories in breast cancer patients, which found that CRF peaked 1 week post-chemotherapy.^[36]^ Based on the pro-inflammatory cytokine theory, studies have shown that inflammatory factors such as IL1-ra, IL-6 and TNF-α as significant indicators of CRF.^[37]^ The decrease in erythrocyte count and hemoglobin level suggest potential involvement of anemia and immune regulation in the mechanisms of CRF.^[38]^ Furthermore, factors such as age, marital status, complications, and other symptoms like anxiety and depression were predictive of fatigue.^[39]^ The severity of symptoms varied across study groups or at different time points within the same study group,^[36]^ underscoring the complexity of the pathogenetic mechanisms and risk factors associated with CRF. This finding is also basically consistent with the high-frequency keywords that appear in this study.

Fourthly, nursing management of CRF has garnered interest over the years. The timeline shows that research on nursing care for patients with CRF did not begin until 2014. Scholars have gradually recognized that CRF is a disease that needs to be managed throughout the process and evaluated repeatedly. The diagnosis and treatment should be divided into 4 stages: screening, preliminary assessment, intervention, and reassessment. Physical activity and psychosocial interventions are currently considered the primary treatments for reducing CRF.^[40]^ Simultaneously, nursing education, mindfulness cognitive therapy, nutrition intervention and acupuncture have proven effective in alleviating CRF in patients with various types of cancer.^[41,42]^ Several studies have conducted meta-analyses on the nursing management of CRF, proposing psychological, social, cognitive and emotional interventions that should be implemented in patients with CRF.^[43,44]^ It is anticipated that the management of CRF will emerge as a significant area of focus in nursing research in the coming years.

The analysis revealed that the most recent keyword with citation bursts that occurred in 2019 was “risk factor.” This coincided with the findings from both the keyword co-occurrence analysis and the timeline view, indicating a consistent research focus on this topic since 2019. The keyword with the highest strength was “surgery,” which emerged in 2016. Scholarly interest in this area has been piqued by the high incidence and severity of CRF observed in cancer patients post-surgery. Notably, CRF is not only a post-surgical complication but also frequently manifests during treatment and persists throughout the disease progression. Consequently, scholarly interest in this keyword diminished rapidly after 1 year. Furthermore, the severity and clinical management of CRF are also research frontiers in this field. It is estimated that between 30% to 70% of patients experience severe CRF, with moderate-to-severe cases often impeding daily activities.^[45]^ Therefore, it is crucial for nurses accurately assess the severity of the symptom of CRF.

### 4.1. Limitations

This study is the first review of CRF in the nursing field, which utilizes systematic and visual analysis of contributing countries, institutions, journals, authors and major topics. However, this study possesses certain limitations that warrant consideration in subsequent studies. First, data were solely sourced from the Web of Science Core Collection in English. Future investigations could broaden the search to encompass additional databases and incorporate an analysis of articles across diverse languages. Second, the information was extracted using machine learning and natural language processing-based bibliometric tools, potentially introducing biases between our results and those reported by studies utilizing alternative bibliometric tools. In the future, we will use a multi-method bibliometric analysis to improve relevant research.

## 5. Conclusion

In this study, CiteSpace was used to analyze the output of CRF in nursing from 2012 to 2021. We obtained a systematic and comprehensive overview of CRF in the nursing field.

Over the past decade, there has been a notable increase in research on CRF in the nursing field has increased. The USA, as a leading nation, not only published the highest number of articles but also maintains close international collaborations. The University of California, San Francisco, recognized as the most prolific institution, and M Christine, the most published author, are both based in the USA. The Cancer Nursing journal stands out with its significant contribution to CRF nursing research. The 4 research hotspots of CRF in the nursing field were identified: high prevalence, adverse outcomes, influential factors, and nursing interventions of CRF. The research frontiers in recent years mainly focus on assessment tools and their different language versions, risk factors and reviews of CRF. Moving forward, fostering research collaborations between hospital nurses and university researchers is pivotal to delve deeper into the mechanisms and nursing management strategies for CRF.

## Acknowledgments

The authors would like to acknowledge all participants and the research support from the School of Nursing, Anhui Medical University.

## Author contributions

**Conceptualization:** Rong Zheng.

**Data curation:** Rong Zheng, Xi Chen, Xiuzhi Xu.

**Formal analysis:** Xi Chen, Xiuzhi Xu.

**Methodology:** Rong Zheng.

**Software:** Rong Zheng.

**Supervision:** Yongxia Song, Wenru Wang, Jingfang Hong.

**Validation:** Xiaodi Ju.

**Visualization:** Rong Zheng.

**Writing – original draft:** Rong Zheng.

**Writing – review & editing:** Yongxia Song, Wenru Wang, Jingfang Hong.
